# Editorial: Safety and Tolerability of Psychotropic Compounds

**DOI:** 10.3389/fnins.2020.00509

**Published:** 2020-06-18

**Authors:** Angel L. Montejo, Mario Lourenço

**Affiliations:** ^1^Faculty of Nursing, IBSAL, University of Salamanca, Salamanca, Spain; ^2^Department of Psychiatry, Hospital da Senhora da Oliveira Guimarães, Guimarães, Portugal

**Keywords:** safety, tolerability, psychotropic adverse effects, hyperprolactinemia, tardive dykinesia, anticholinegic, antipsychotic

One of the greatest advances in modern psychiatry was undoubtedly the discovery of psychopharmacology. It has become a powerful therapeutic tool to fight against mental illnesses since then. Nevertheless, a new stage emerged seeking effectiveness including tolerability and safety and not just efficacy.

A special challenge includes avoiding relapses while increasing safety and finding optimal tolerability in the medium and long term. Unfortunately, some side effects may remain unnoticed when not detected in short clinical trials such is the case of the neurological, metabolic, or endocrinological side effects of some antipsychotics such as tardive dyskinesia, metabolic syndrome, or iatrogenic hyperprolactinemia. In the case of serotonergic antidepressants (SSRIS), sexual dysfunction, a relevant but almost unnoticed adverse effect in clinical trials, has been subsequently identified by patients and clinicians as the most frequent adverse effect in the medium and long term. Unfortunately, its frequency remains highly under rated (5–15%) when compared with clinical practice epidemiological data (50–70%) (Clayton et al., 2016; Montejo et al., 2018, 2019).

Pharmacovigilance is essential in the new era of psychotropic drugs. The majority of safety and tolerability data have been obtained after longitudinal prescriptions and through cohort studies at naturalistic settings. Tolerability and safety are crucial, mainly in sensitive populations such as children and the elderly, both to avoid unexpected effects that can lead to serious events and to ensure compliance and quality of life.

More and more doctors, in general, and psychiatrists, in particular, often care for patients who suffer from a chronic pluripathology. In these patients, one of the great challenges of clinical practice is related to polypharmacy and drug interactions causing situations of difficult clinical management.

Among psychotropic drugs, antipsychotics are an excellent working tool for doctors. In addition to the incisive action on the main symptoms of Schizophrenia, atypical antipsychotics also have the ability to promote neurogenesis, which in the hippocampus may be responsible for some of its antidepressant effects. The hippocampus plays an important role in the process of learning and memorizing, knowledge, regularization of mood, and response to stress. As far as neurogenesis is concerned, we have seen that in schizophrenia there is, before the start of treatment, a neurotropin breakdown such as BDNF (brain-derived neurotrophic factor) and NGF (nervous growth factor) (Nandra and Agius, 2012).

One of the uncommon but serious event related to the classical antipsychotics is Tardive Dyskinesia (TDK). However, its relationship with atypical dopamine D2 partial agonist, such as aripiprazole, is very scarce although its therapeutic approach is essential to improve quality of life, prevent neurological deterioration and functionality. Theoretically, long-term exposure of D2 receptors to aripiprazole could cause irreversible hypersensitivity although it is extremely infrequent. The combination treatment that improved this aripiprazole-related TDK with tetrabenazine, clozapine, and botulinum toxin can be an opportunity for patients considering the pathophysiological mechanisms underlying this pathology that can become very invalidating. The main essential pharmacological property of tetrabenazine is the reduction of monoamines (dopamine, serotonin, and noradrenaline) in the CNS, particularly dopamine, and it causes a reversible inhibition of the activity of vesicle monoamine transporter. Botulinum toxin shows a paralyzing action that binds to the presynaptic nerve terminals inhibiting the secretion of acetylcholine. Even though clozapine has some limitations (such as agranulocytosis), it can be an effective alternative in the treatment of irreversible TDK. The combination of these three agents, never previously used together, was useful in a patient who did not respond to the isolated use of each of them (Aguilar et al.).

Hyperprolactinemia is a common consequence of treatment with some antipsychotics, mainly risperidone, paliperidone, and amisulpride but less frequently related to olanzapine. Two cases of iatrogenic hyperprolactinemia are described by olanzapine that are successfully treated switching to aripiprazole (Cabral Barata et al.). Hyperprolactinemia can be divided according to its severity: mild (50 ng/ml), moderate (51–75 ng/ml), and severe (>100 ng/ml) showing relevant clinical consequences in the short, medium and long term, such as bone mineralization, osteopenia and osteoporosis, increasing cardiovascular risk, and deteriorating sexual function. These symptoms have been linked to a lack of treatment adherence in young and sexually active patients needing consensus recommendations and clinical guidelines for their treatment (Montejo et al., 2017). In diseases such as Schizophrenia, the rate of non-adherence to treatments can reach 60%. The consequences are inevitable: multiple relapses and subsequent exacerbations; great use of healthcare resources; increase risk of suicide attempts; long term symptoms and disability; decrease in treatment response (Higashi et al., 2013). The patient's lack of compliance is due to the lack of insight regarding the disease, but it also arises due to the side effects of antipsychotics.

Geriatric population requires special monitoring in the prescription of psychotropic compounds taking into account aspects little considered as the anticholinergic effects. Anticholinergic drugs block acetylcholine neurotransmission, which is involved in the central and peripheral functions. The adverse effects on the central nervous system include cognitive impairment, increase of neurodegenerative processes, and the presence of psychotic or confusional symptoms. Additionally, peripheral effects such as dry mouth, urinary retention, constipation, paralytic ileus, increased heart rate, and blurred vision could severely affect the safety of the patients. The general recommendation would be to exercise extreme caution with agents with important anticholinergic effects including tricyclic antidepressants, antipsychotics (clozapine, olanzapine), carbamazepine, benztropine, and biperiden. In any case, systematic monitoring of these potential adverse effects on the elderly is essential in order not to compromise their quality of life and safety (López-Álvarez et al.).

Complementing the above, it is important to pay attention to the metabolites of psychotropic compounds since they do not always possess the same pharmacological properties as their predecessors. Quetiapine is widely used in delusion patients and in the elderly with delirium, but the appearing of delirium caused by the use of quetiapine in the elderly it is uncommon. The pathophysiology of delirium remains poorly understood yet involving multifactorial interactions between different risk factors. Many neurotransmitters are potentially implicated in the pathophysiology of delirium, but cholinergic dysfunction is one of the most frequently linked to delirium pathophysiology. The potential anticholinergic effects of quetiapine, mediated primarily through its metabolite norquetiapine, could theoretically be linked to this adverse effect (Almeida et al.). Norquetiapine is the major active metabolite of quetiapine and is produced by the action of isoenzyme CYP34A in cytochrome P450 so the determination of possible drug interactions is essential. In the elderly, the clinical learning is that the combination of several antipsychotics, antidepressants, and benzodiazepines, all with anticholinergic effect, can be linked to some confusional pictures. It could be avoided by reducing the use of psychotropics to a minimum, when possible alone.

Finally, the safety and tolerability of psychotropic compounds should always be present as an essential element in the prescription process. Monitoring of adverse effects should be aimed at identifying those that may be asymptomatic in the short, medium and long term (such as hyperprolactinemia) as they may go unnoticed and compromise the quality of life and safety.

## Author Contributions

All authors listed have made a substantial, direct and intellectual contribution to the work, and approved it for publication.

## Conflict of Interest

AM has received consultancy fees or honoraria/research grants in the last 5 years from Eli Lilly, Forum Pharmaceuticals, Rovi, Servier, Lundbeck, Otsuka, Janssen Cilag, Pfizer, Roche, Instituto de Salud Carlos III, Junta de Castilla y León, and Osakidetxa. ML declares no competing interests with regards to the Research Topic subject.
